# The genome sequence of a drosophilid fruit fly, *Drosophila limbata* von Roser 1840

**DOI:** 10.12688/wellcomeopenres.22584.1

**Published:** 2024-07-10

**Authors:** Darren J. Obbard, Darwin Tree of Life Barcoding collective, Wellcome Sanger Institute Tree of Life Management, Samples and Laboratory team, Wellcome Sanger Institute Scientific Operations: Sequencing Operations, Wellcome Sanger Institute Tree of Life Core Informatics team, Tree of Life Core Informatics collective, Darwin Tree of Life Consortium

**Affiliations:** 1 Institute of Ecology and Evolution, The University of Edinburgh, Edinburgh, Scotland, UK

**Keywords:** Drosophila limbata, drosophilid fruit fly, genome sequence, chromosomal, Diptera

## Abstract

We present a genome assembly from an individual male *Drosophila limbata* (drosophilid fruit fly; Arthropoda; Insecta; Diptera; Drosophilidae). The genome sequence is 233.5 megabases in span. Most of the assembly is scaffolded into 6 chromosomal pseudomolecules. The mitochondrial genome has also been assembled and is 16.09 kilobases in length.

## Species taxonomy

Eukaryota; Opisthokonta; Metazoa; Eumetazoa; Bilateria; Protostomia; Ecdysozoa; Panarthropoda; Arthropoda; Mandibulata; Pancrustacea; Hexapoda; Insecta; Dicondylia; Pterygota; Neoptera; Endopterygota; Diptera; Brachycera; Muscomorpha; Eremoneura; Cyclorrhapha; Schizophora; Acalyptratae; Ephydroidea; Drosophilidae; Drosophilinae; Drosophilini; Drosophila; *Drosophila*; *quinaria* group; *Drosophila limbata* von Roser 1840 (NCBI:txid42028).

## Background

**Figure 1.**
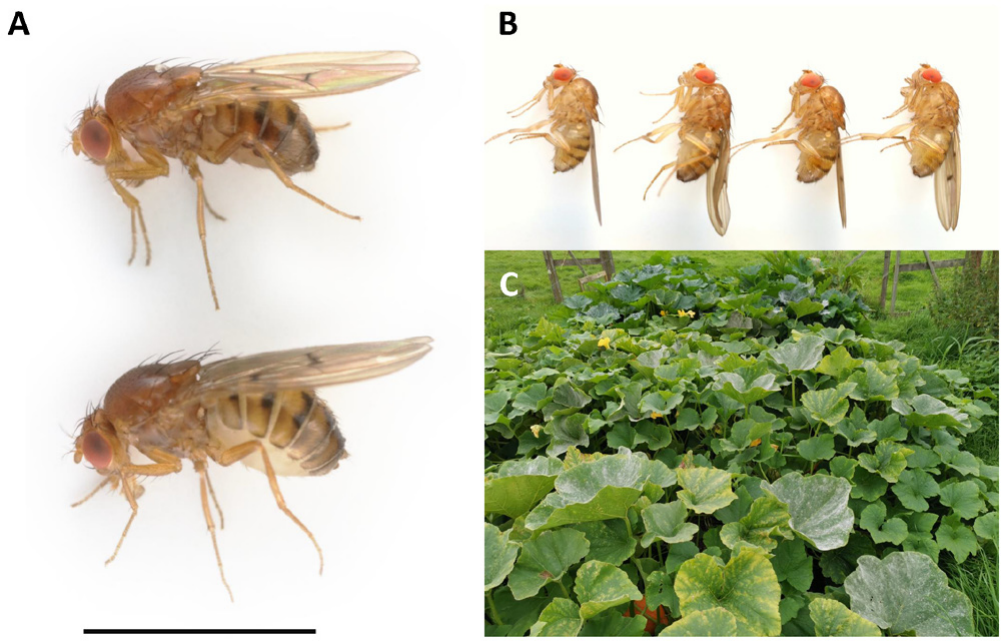
*Drosophila limbata* specimens. **A:** Wild-collected male (above) and female (below) *Drosophila limbata* presented with a 3 mm scale bar. **B:** The four lab-reared brothers selected for sequencing: specimen ID SAN00001918, ToLID idDroLimb2 (second from left) used for PacBio sequencing, specimen ID SAN00001919, ToLID idDroLimb3 (second from right) used for Hi-C sequencing, and specimen ID SAN00001920, ToLID idDroLimb4 (right) used for RNA sequencing. **C:** The vegetable patch from which the mother of the sequenced flies was collected on 2021-09-05 (Cherry Gardens Farm, East Sussex, England; 51.0994 N, 0.1639 E).

*Drosophila limbata* von Roser 1840 is a medium sized ( *ca.* 3.0–3.5 mm) yellowish-brown drosophilid ‘fruit fly’ ( Figure 1A and 1B). It is one of around 30 British and Irish species of *Drosophila* ( Chandler, 2023), and is a member of the *quinaria* species group within the subgenus *Drosophila* ( Bächli *et al.*, 2004). Flies are superficially similar in appearance to their close relative *Drosophila kuntzei* ( Bächli *et al.*, 2004), but can be separated on the shape of the abdominal bands, by dissection of the terminalia, and (in wild flies) by their overall darker brown colouration ( Figure 1A). Unlike most other members of the *quinaria* group, which are predominantly fungus specialists ( Scott Chialvo *et al.*, 2019), *D. limbata* uses decaying plant matter as a substrate, including several species of Cucurbitaceae and Apiaceae ( Hummel *et al.*, 1979; Offenberger & Klarenberg, 1992; van Alphen *et al.*, 1991). Although *Drosophila limbata* have been maintained in laboratory culture, the species seems to have been remarkably little studied, with just a handful of papers discussing such disparate topics as population dynamics ( Hummel *et al.*, 1979), parasitism ( Gillis & Hardy, 1997; van Alphen *et al.*, 1991), alcohol tolerance ( Mercot *et al.*, 1994), and courtship song ( Neems *et al.*, 1997).

In nature, *D. limbata* is broadly distributed across the palearctic, from the West of Ireland to the East of Russia, and from Crete in the south to central Finland to the north ( Bächli, 2024). Relatively few records are available for the UK ( GBIF Secretariat, 2024), and the species was not reported either from Scotland by Basden in 1950–52 (43,629 flies examined; Basden (1955)) or from a survey of Southern England by Dyson-Hudson in 1952–53 (18,535 flies examined in the survey, although a total of eight *D. limbata* were reported to have been caught separately; Dyson-Hudson (1954)). Nevertheless, the adults can be seen across much of the year ( GBIF Secretariat, 2024), and the species is not reported to be threatened. It thus seems likely that the scarcity of UK records reflects the challenge of identification, and the failure of these flies to come to fruit baits.

Here we present a chromosomally complete genome sequence for *Drosophila limbata*, derived from the DNA of three male offspring of a wild female that was collected from courgette and squash plants at Cherry Gardens Farm, East Sussex, as part of the Darwin Tree of Life Project. This genome sequence will help to resolve relationships among the Drosophilidae and will further build on the value of this family as a model clade for comparative genomics and molecular evolution. This project is a collaborative effort to sequence all named eukaryotic species in the Atlantic Archipelago of Britain and Ireland.

## Genome sequence report

The genome was sequenced from a male *Drosophila limbata* ( Figure 1) reared at the Institute of Ecology and Evolution, University of Edinburgh. A total of 107-fold coverage in Pacific Biosciences single-molecule HiFi long reads was generated. Primary assembly contigs were scaffolded with chromosome conformation Hi-C data. Manual assembly curation corrected 28 missing joins or mis-joins and removed 5 haplotypic duplications, reducing the scaffold number by 1.92%, and decreasing the scaffold N50 by 6.57%.

**Figure 2.**
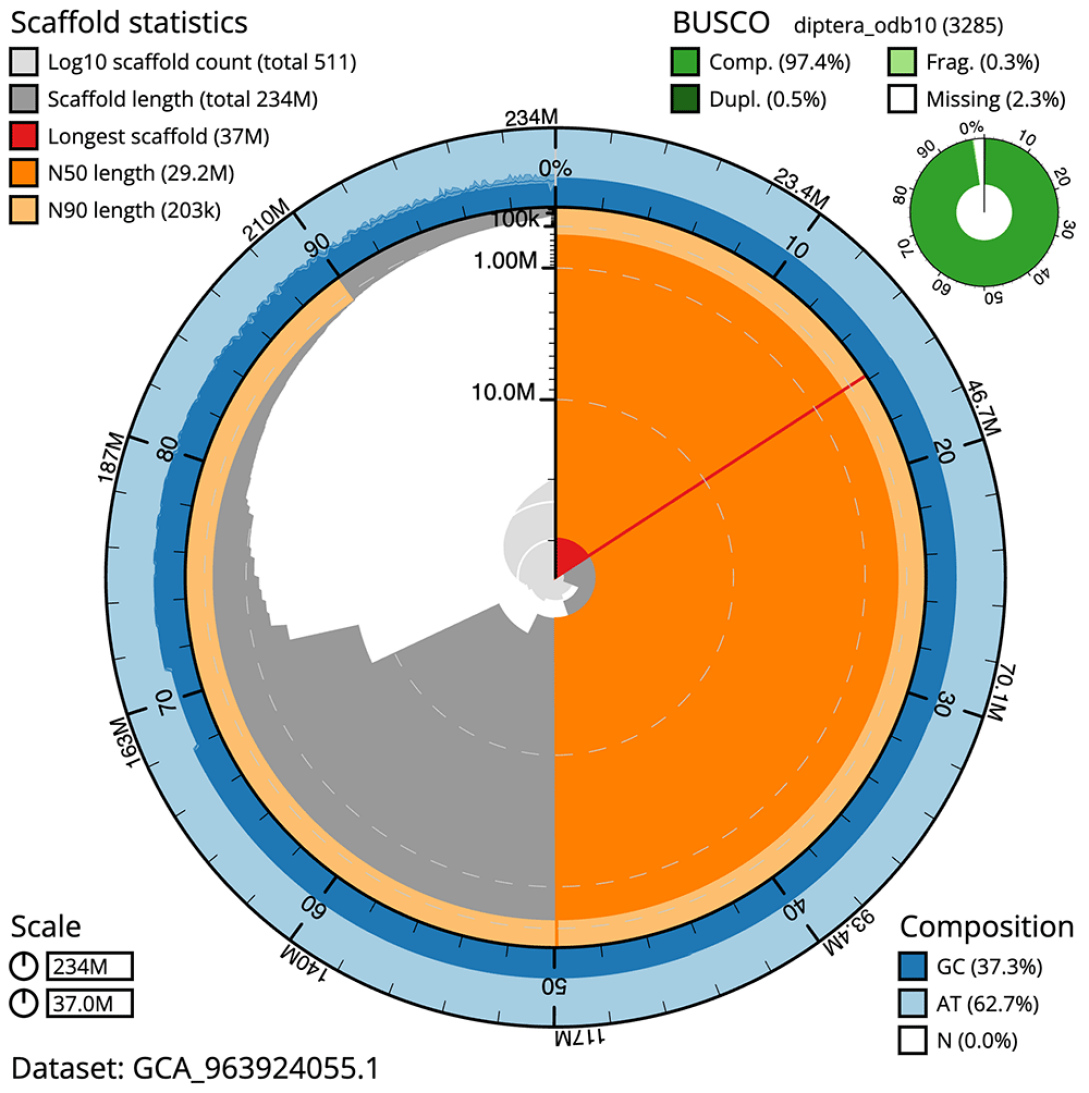
Genome assembly of *Drosophila limbata*, idDroLimb2.1: metrics. The BlobToolKit snail plot shows N50 metrics and BUSCO gene completeness. The main plot is divided into 1,000 size-ordered bins around the circumference with each bin representing 0.1% of the 233,538,449 bp assembly. The distribution of scaffold lengths is shown in dark grey with the plot radius scaled to the longest scaffold present in the assembly (37,002,035 bp, shown in red). Orange and pale-orange arcs show the N50 and N90 scaffold lengths (29,161,486 and 202,746 bp), respectively. The pale grey spiral shows the cumulative scaffold count on a log scale with white scale lines showing successive orders of magnitude. The blue and pale-blue area around the outside of the plot shows the distribution of GC, AT and N percentages in the same bins as the inner plot. A summary of complete, fragmented, duplicated and missing BUSCO genes in the diptera_odb10 set is shown in the top right. An interactive version of this figure is available at https://blobtoolkit.genomehubs.org/view/Drosophila_limbata/dataset/GCA_963924055.1/snail.

**Figure 3.**
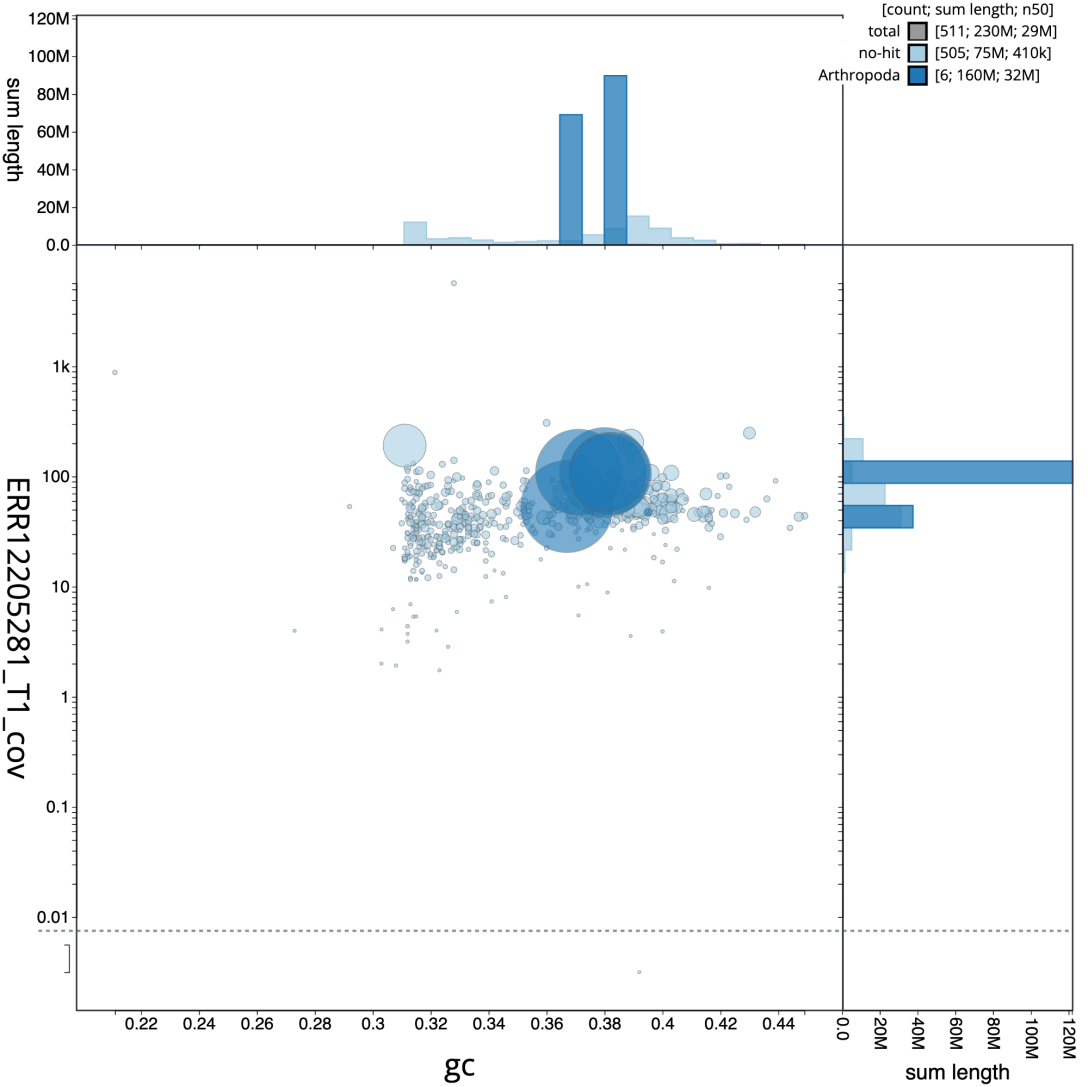
Genome assembly of *Drosophila limbata*, idDroLimb2.1: BlobToolKit GC-coverage plot. Sequences are coloured by phylum. Circles are sized in proportion to sequence length. Histograms show the distribution of sequence length sum along each axis. An interactive version of this figure is available at https://blobtoolkit.genomehubs.org/view/Drosophila_limbata/dataset/GCA_963924055.1/blob.

**Figure 4.**
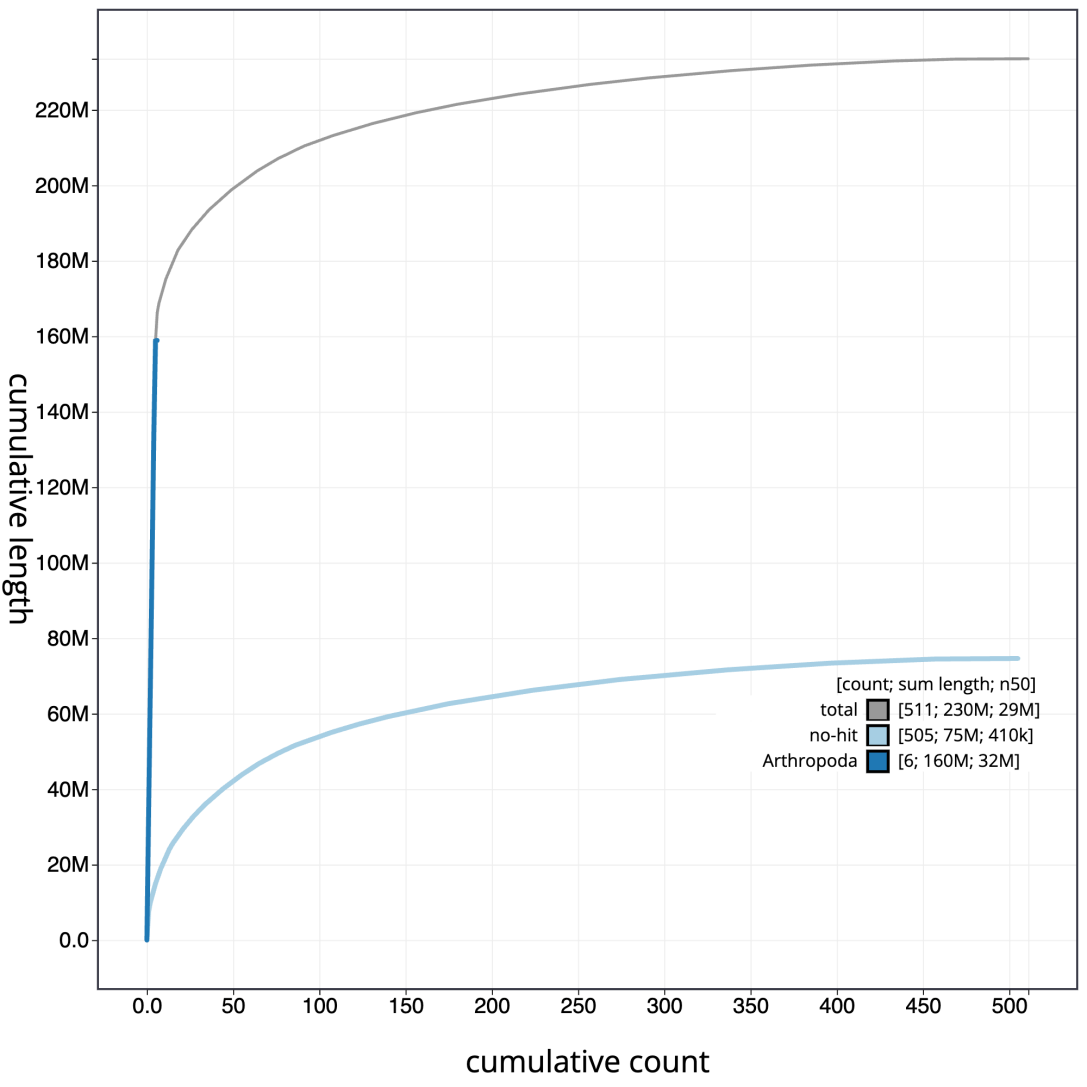
Genome assembly of *Drosophila limbata*, idDroLimb2.1: BlobToolKit cumulative sequence plot. The grey line shows cumulative length for all sequences. Coloured lines show cumulative lengths of sequences assigned to each phylum using the buscogenes taxrule. An interactive version of this figure is available at https://blobtoolkit.genomehubs.org/view/Drosophila_limbata/dataset/GCA_963924055.1/cumulative.

**Figure 5.**
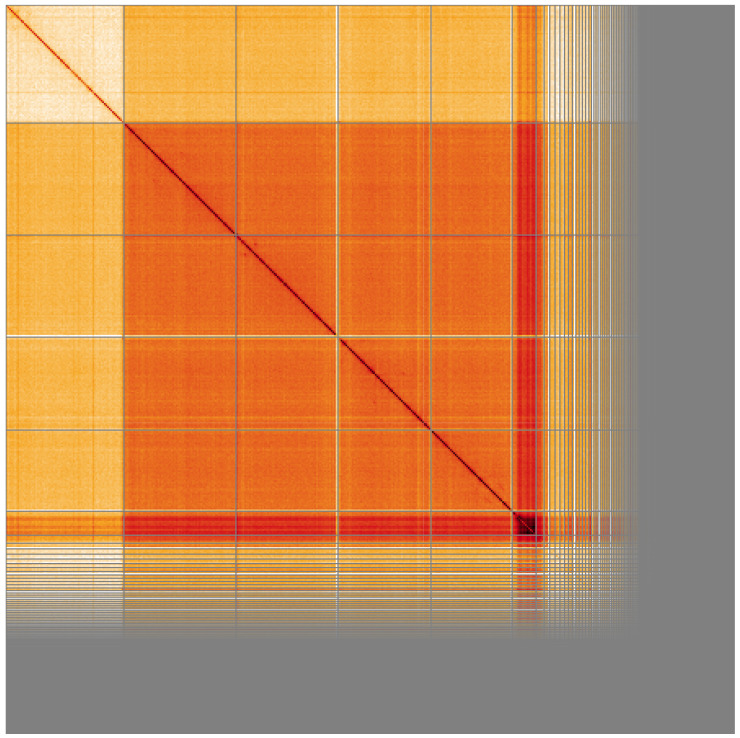
Genome assembly of *Drosophila limbata*, idDroLimb2.1: Hi-C contact map of the idDroLimb2.1 assembly, visualised using HiGlass. Chromosomes are shown in order of size from left to right and top to bottom. An interactive version of this figure may be viewed at https://genome-note-higlass.tol.sanger.ac.uk/l/?d=A6WVNgRTTCy8cVHqR08cvA.

The final assembly has a total length of 233.5 Mb in 510 sequence scaffolds with a scaffold N50 of 29.2 Mb ( Table 1). The snail plot in Figure 2 provides a summary of the assembly statistics, while the distribution of assembly scaffolds on GC proportion and coverage is shown in Figure 3. The cumulative assembly plot in Figure 4 shows curves for subsets of scaffolds assigned to different phyla. Most (71.2%) of the assembly sequence was assigned to 6 chromosomal-level scaffolds, representing 5 autosomes and the X sex chromosome. Chromosome-scale scaffolds confirmed by the Hi-C data are named in order of size ( Figure 5; Table 2). The X chromosome was identified based on PacBio read coverage. We expected to find a Y chromosome, but this could not be identified and is likely in the unplaced contigs. The order and orientation of contigs along Chromosome 6 between 1.6 Mb and 7.4 Mb is uncertain. While not fully phased, the assembly deposited is of one haplotype. Contigs corresponding to the second haplotype have also been deposited. The mitochondrial genome was also assembled and can be found as a contig within the multifasta file of the genome submission.

**Table 1.** Genome data for *Drosophila limbata*, idDroLimb2.1.

Project accession data		
Assembly identifier	idDroLimb2.1	
Species	*Drosophila limbata*	
Specimen	idDroLimb2	
NCBI taxonomy ID	42028	
BioProject	PRJEB68012	
BioSample ID	SAMEA12110471	
Isolate information	idDroLimb2: whole organism (genome sequence) idDroLimb3: whole organism (Hi-C sequencing) idDroLimb4: whole organism (RNA sequencing)	
Assembly metrics *		*Benchmark*
Consensus quality (QV)	59.6	*≥ 50*
*k*-mer completeness	100.0%	*≥ 95%*
BUSCO **	C:97.4%[S:96.9%,D:0.5%], F:0.3%,M:2.3%,n:3,285	*C ≥ 95%*
Percentage of assembly mapped to chromosomes	71.2%	*≥ 95%*
Sex chromosomes	X	*localised * *homologous pairs*
Organelles	Mitochondrial genome: 16.09 kb	*complete single * *alleles*
Raw data accessions		
PacificBiosciences Sequel IIe	ERR12205281	
Hi-C Illumina	ERR12245608, ERR12245609	
PolyA RNA-Seq Illumina	ERR12708753	
Genome assembly		
Assembly accession	GCA_963924055.1	
*Accession of alternate haplotype*	GCA_963924035.1	
Span (Mb)	233.5	
Number of contigs	733	
Contig N50 length (Mb)	1.0	
Number of scaffolds	510	
Scaffold N50 length (Mb)	29.2	
Longest scaffold (Mb)	37.0	

* Assembly metric benchmarks are adapted from column VGP-2020 of “Table 1: Proposed standards and metrics for defining genome assembly quality” from Rhie *et al.* (2021). ** BUSCO scores based on the diptera_odb10 BUSCO set using version v5.4.3. C = complete [S = single copy, D = duplicated], F = fragmented, M = missing, n = number of orthologues in comparison. A full set of BUSCO scores is available at https://blobtoolkit.genomehubs.org/view/Drosophila_limbata/dataset/GCA_963924055.1/busco.

**Table 2.** Chromosomal pseudomolecules in the genome assembly of *Drosophila limbata*, idDroLimb2.

INSDC accession	Chromosome	Length (Mb)	GC%
OZ001352.1	1	35.13	38.0
OZ001353.1	2	32.02	37.0
OZ001354.1	3	29.16	38.0
OZ001355.1	4	25.45	38.0
OZ001356.1	5	7.52	31.0
OZ001351.1	X	37.0	36.5
OZ001357.1	MT	0.02	22.0

The estimated Quality Value (QV) of the final assembly is 59.6 with *k*-mer completeness of 100.0%, and the assembly has a BUSCO v completeness of 97.4% (single = 96.9%, duplicated = 0.5%), using the diptera_odb10 reference set ( *n* = 3,285).

Metadata for specimens, BOLD barcode results, spectra estimates, sequencing runs, contaminants and pre-curation assembly statistics are given at https://links.tol.sanger.ac.uk/species/42028.

## Methods

### Sample acquisition and nucleic acid extraction

The *Drosophila limbata* specimens used in the genome assembly were first-generation male progeny from a wild-collected female. The mother was collected from a vegetable patch (Cherry Gardens Farm, East Sussex, England; 51.0994 N, 0.1639 E) on 2021-09-05. The sequenced flies were reared on a standard laboratory banana *Drosophila* medium ( https://figshare.com/articles/figure/Drosophila_Media_Recipes/21590724). *Drosophila limbata* specimen ID SAN00001918 (ToLID idDroLimb2) was used for PacBio DNA sequencing, specimen ID SAN00001919 (ToLID idDroLimb3) was used for Hi-C sequencing, and specimen ID SAN00001920 (ToLID idDroLimb4) was used for RNA sequencing.

The workflow for high molecular weight (HMW) DNA extraction at the Wellcome Sanger Institute (WSI) Tree of Life Core Laboratory includes a sequence of core procedures: sample preparation; sample homogenisation, DNA extraction, fragmentation, and clean-up. The idDroLimb2 sample was weighed and dissected on dry ice ( Jay *et al.*, 2023), and tissue was homogenised using a PowerMasher II tissue disruptor ( Denton *et al.*, 2023a). HMW DNA was extracted using the Manual MagAttract v1 protocol ( Strickland *et al.*, 2023b). DNA was sheared into an average fragment size of 12–20 kb in a Megaruptor 3 system with speed setting 30 ( Todorovic *et al.*, 2023). Sheared DNA was purified by solid-phase reversible immobilisation ( Strickland *et al.*, 2023a): in brief, the method employs a 1.8X ratio of AMPure PB beads to sample to eliminate shorter fragments and concentrate the DNA. The concentration of the sheared and purified DNA was assessed using a Nanodrop spectrophotometer and Qubit Fluorometer and Qubit dsDNA High Sensitivity Assay kit. Fragment size distribution was evaluated by running the sample on the FemtoPulse system.

RNA was extracted from the idDroLimb4 sample in the Tree of Life Laboratory at the WSI using the RNA Extraction: Automated MagMax™ *mir*Vana protocol ( do Amaral *et al.*, 2023). The RNA concentration was assessed using a Nanodrop spectrophotometer and a Qubit Fluorometer using the Qubit RNA Broad-Range Assay kit. Analysis of the integrity of the RNA was done using the Agilent RNA 6000 Pico Kit and Eukaryotic Total RNA assay.

Protocols developed by the WSI Tree of Life laboratory are publicly available on protocols.io ( Denton *et al.*, 2023b).

### Sequencing

Pacific Biosciences HiFi circular consensus DNA sequencing libraries were constructed according to the manufacturers’ instructions. Poly(A) RNA-Seq libraries were constructed using the NEB Ultra II RNA Library Prep kit. DNA and RNA sequencing was performed by the Scientific Operations core at the WSI on Pacific Biosciences Sequel IIe (HiFi) and Illumina NovaSeq 6000 (RNA-Seq) instruments. Hi-C data were also generated from specimen idDroLimb3 using the Arima2 kit and sequenced on the Illumina NovaSeq 6000 instrument.

### Genome assembly and curation

Assembly was carried out with Hifiasm ( Cheng *et al.,* 2021) and haplotypic duplication was identified and removed with purge_dups ( Guan *et al.,* 2020). The assembly was then scaffolded with Hi-C data ( Rao *et al.*, 2014) using YaHS ( Zhou *et al.*, 2023). The assembly was checked for contamination and corrected using the TreeVal pipeline ( Pointon *et al.*, 2023). Manual curation was performed using JBrowse2 ( Diesh *et al.,* 2023), HiGlass ( Kerpedjiev *et al.*, 2018) and PretextView ( Harry, 2022). The mitochondrial genome was assembled using MitoHiFi ( Uliano-Silva *et al.,* 2023), which runs MitoFinder ( Allio *et al.*, 2020) and uses these annotations to select the final mitochondrial contig and to ensure the general quality of the sequence. The mitochondrial reference was *Drosophila suzukii* (NC_060762.1).

### Final assembly evaluation

The final assembly was post-processed and evaluated with the three Nextflow ( Di Tommaso *et al.*, 2017) DSL2 pipelines “sanger-tol/readmapping” ( Surana *et al.*, 2023a), “sanger-tol/genomenote” ( Surana *et al.*, 2023b), and “sanger-tol/blobtoolkit” ( Muffato *et al.*, 2024). The pipeline sanger-tol/readmapping aligns the Hi-C reads with bwa-mem2 ( Vasimuddin *et al.*, 2019) and combines the alignment files with SAMtools ( Danecek *et al.*, 2021). The sanger-tol/genomenote pipeline transforms the Hi-C alignments into a contact map with BEDTools ( Quinlan & Hall, 2010) and the Cooler tool suite ( Abdennur & Mirny, 2020), which is then visualised with HiGlass ( Kerpedjiev *et al.*, 2018). It also provides statistics about the assembly with the NCBI datasets ( Sayers *et al.*, 2024) report, computes *k*-mer completeness and QV consensus quality values with FastK and MerquryFK, and a completeness assessment with BUSCO ( Manni *et al.*, 2021).

The sanger-tol/blobtoolkit pipeline is a Nextflow port of the previous Snakemake Blobtoolkit pipeline ( Challis *et al.*, 2020). It aligns the PacBio reads with SAMtools and minimap2 ( Li, 2018) and generates coverage tracks for regions of fixed size. In parallel, it queries the GoaT database ( Challis *et al.*, 2023) to identify all matching BUSCO lineages to run BUSCO ( Manni *et al.*, 2021). For the three domain-level BUSCO lineage, the pipeline aligns the BUSCO genes to the Uniprot Reference Proteomes database ( Bateman *et al.*, 2023) with DIAMOND ( Buchfink *et al.*, 2021) blastp. The genome is also split into chunks according to the density of the BUSCO genes from the closest taxonomically lineage, and each chunk is aligned to the Uniprot Reference Proteomes database with DIAMOND blastx. Genome sequences that have no hit are then chunked with seqtk and aligned to the NT database with blastn ( Altschul *et al.*, 1990). All those outputs are combined with the blobtools suite into a blobdir for visualisation.

All three pipelines were developed using the nf-core tooling ( Ewels *et al.*, 2020), use MultiQC ( Ewels *et al.*, 2016), and make extensive use of the Conda package manager, the Bioconda initiative ( Grüning *et al.*, 2018), the Biocontainers infrastructure ( da Veiga Leprevost *et al.*, 2017), and the Docker ( Merkel, 2014) and Singularity ( Kurtzer *et al.*, 2017) containerisation solutions.

Table 3 contains a list of relevant software tool versions and sources.

**Table 3.** Software tools: versions and sources.

Software tool	Version	Source
BEDTools	2.30.0	https://github.com/arq5x/bedtools2
Blast	2.14.0	ftp://ftp.ncbi.nlm.nih.gov/blast/executables/blast+/
BlobToolKit	4.3.7	https://github.com/blobtoolkit/blobtoolkit
BUSCO	5.4.3	https://gitlab.com/ezlab/busco
BUSCO	5.4.3 and 5.5.0	https://gitlab.com/ezlab/busco
bwa-mem2	2.2.1	https://github.com/bwa-mem2/bwa-mem2
Cooler	0.8.11	https://github.com/open2c/cooler
DIAMOND	2.1.8	https://github.com/bbuchfink/diamond
fasta_ windows	0.2.4	https://github.com/tolkit/fasta_windows
FastK	427104ea91c78c3b8b8b49f1a7d6bbeaa869ba1c	https://github.com/thegenemyers/FASTK
GoaT CLI	0.2.5	https://github.com/genomehubs/goat-cli
Hifiasm	0.16.1-r375	https://github.com/chhylp123/hifiasm
HiGlass	1.11.6	https://github.com/higlass/higlass
HiGlass	44086069ee7d4d3f6f3f0012569789ec138f42b84aa4435 7826c0b6753eb28de	https://github.com/higlass/higlass
MerquryFK	d00d98157618f4e8d1a9190026b19b471055b22e	https://github.com/thegenemyers/MERQURY.FK
MitoHiFi	2	https://github.com/marcelauliano/MitoHiFi
MultiQC	1.14, 1.17, and 1.18	https://github.com/MultiQC/MultiQC
NCBI Datasets	15.12.0	https://github.com/ncbi/datasets
Nextflow	23.04.0-5857	https://github.com/nextflow-io/nextflow
PretextView	0.2	https://github.com/wtsi-hpag/PretextView
purge_dups	1.2.3	https://github.com/dfguan/purge_dups
samtools	1.16.1, 1.17, and 1.18	https://github.com/samtools/samtools
sanger-tol/ genomenote	1.1.1	https://github.com/sanger-tol/genomenote
sanger-tol/ readmapping	1.2.1	https://github.com/sanger-tol/readmapping
Seqtk	1.3	https://github.com/lh3/seqtk
Singularity	3.9.0	https://github.com/sylabs/singularity
TreeVal	1.0.0	https://github.com/sanger-tol/treeval
YaHS	1.1a.2	https://github.com/c-zhou/yahs

### Wellcome Sanger Institute – Legal and Governance

The materials that have contributed to this genome note have been supplied by a Darwin Tree of Life Partner. The submission of materials by a Darwin Tree of Life Partner is subject to the **‘Darwin Tree of Life Project Sampling Code of Practice’**, which can be found in full on the Darwin Tree of Life website here. By agreeing with and signing up to the Sampling Code of Practice, the Darwin Tree of Life Partner agrees they will meet the legal and ethical requirements and standards set out within this document in respect of all samples acquired for, and supplied to, the Darwin Tree of Life Project. 

Further, the Wellcome Sanger Institute employs a process whereby due diligence is carried out proportionate to the nature of the materials themselves, and the circumstances under which they have been/are to be collected and provided for use. The purpose of this is to address and mitigate any potential legal and/or ethical implications of receipt and use of the materials as part of the research project, and to ensure that in doing so we align with best practice wherever possible. The overarching areas of consideration are:

• Ethical review of provenance and sourcing of the material

• Legality of collection, transfer and use (national and international)

Each transfer of samples is further undertaken according to a Research Collaboration Agreement or Material Transfer Agreement entered into by the Darwin Tree of Life Partner, Genome Research Limited (operating as the Wellcome Sanger Institute), and in some circumstances other Darwin Tree of Life collaborators.

## Data Availability

European Nucleotide Archive: *Drosophila limbata*. Accession number PRJEB68012; https://identifiers.org/ena.embl/PRJEB68012 ( Wellcome Sanger Institute, 2023). The genome sequence is released openly for reuse. The *Drosophila limbata* genome sequencing initiative is part of the Darwin Tree of Life (DToL) project. All raw sequence data and the assembly have been deposited in INSDC databases. The genome will be annotated using available RNA-Seq data and presented through the Ensembl pipeline at the European Bioinformatics Institute. Raw data and assembly accession identifiers are reported in Table 1.

## Author information

Members of the Darwin Tree of Life Barcoding collective are listed here: https://doi.org/10.5281/zenodo.4893703.

Members of the Wellcome Sanger Institute Tree of Life Management, Samples and Laboratory team are listed here: https://doi.org/10.5281/zenodo.10066175.

Members of Wellcome Sanger Institute Scientific Operations: Sequencing Operations are listed here: https://doi.org/10.5281/zenodo.10043364.

Members of the Wellcome Sanger Institute Tree of Life Core Informatics team are listed here: https://doi.org/10.5281/zenodo.10066637.

Members of the Tree of Life Core Informatics collective are listed here: https://doi.org/10.5281/zenodo.5013541.

Members of the Darwin Tree of Life Consortium are listed here: https://doi.org/10.5281/zenodo.4783558.
