# Supplementary material for: The genome sequence of a drosophilid fruit fly, Drosophila limbata von Roser 1840
Source: Wellcome Open Res. 2024 Jul 10;9:365. [Version 1] doi: 10.12688/wellcomeopenres.22584.1 (PMC11369588; doi:10.12688/wellcomeopenres.22584.1)
Supplement: Peer review history

# Reviewer report: Reviewer response for version 1

**DOI:** 10.21956/wellcomeopenres.24882.r94748

**Published:** 2024-09-17

**Authors:** Anthony Bayega

**Affiliations:** 1 Technical development, Canada's Michael Smith Genome Sciences Centre, Vancouver, British Columbia, Canada

Obbard et al. provide an overview description of the genome of *Drosophila limbata* which they sequenced and assembled. I commend them for their efforts. My comments follow below:

 1. In the background, the authors provide a brief description of the organism and its geographical distribution.

 2. In Figure 3, the label on the Y-axis which reads “ERR1220…” is completely meaningless. This should be changed to something meaningful to help the reader make sense of this figure.

 3. The authors mention that ‘It aligns the PacBio reads with SAMtools and minimap2..’. For the sake of correctness, please verify that indeed SAMtools was used for alignment and if needed, please edit this statement to accurately and correctly reflect what was done and what was used.

 4. there seems to be a small typing error where it says “taxonomically lineage, and …” Please check and correct that statement if needed.

 Overall, the authors provide a high-quality genome and also assign much of it to chromosomes. Although much work remains to order the scaffolds and complete the gaps and also structurally and functionally annotate the genome, the current work will indeed be valuable to the whole community. I therefore recommend the indexing of this genome so that this resource becomes widely accessible to the scientific community.

Are sufficient details of methods and materials provided to allow replication by others?

Yes

Is the rationale for creating the dataset(s) clearly described?

Yes

Are the datasets clearly presented in a useable and accessible format?

Yes

Are the protocols appropriate and is the work technically sound?

Yes

Reviewer Expertise:

Genomics and molecular biology

I confirm that I have read this submission and believe that I have an appropriate level of expertise to confirm that it is of an acceptable scientific standard, however I have significant reservations, as outlined above.

# Reviewer report: Reviewer response for version 1

**DOI:** 10.21956/wellcomeopenres.24882.r94744

**Published:** 2024-09-02

**Authors:** Qingsong Zhou

**Affiliations:** 1 Chinese Academy of Sciences, Beijing, China

Obbard et al. present a genome assembly of *D. limbata* using Pacific HIFI long reads. The background of the species *D. limbata* is well described. Additionally, the chromosomes were scaffolded with Hi-C data into five autosomes and one sex chromosome (X). The genome size falls within the range of Drosophila genomes (130–257 Mb), and the scaffold N50 of 29.2 Mb indicates that the genome assembly is of high quality. The methodological aspects of this paper are described in detail.

 However, I find the presentation of the Hi-C contact map in Figure 5 unclear, making it difficult to distinguish the five autosomes. Furthermore, it may be beneficial to combine Figures 2-4 into a single figure to enhance the manuscript's conciseness and clarity.

Are sufficient details of methods and materials provided to allow replication by others?

Yes

Is the rationale for creating the dataset(s) clearly described?

Yes

Are the datasets clearly presented in a useable and accessible format?

Yes

Are the protocols appropriate and is the work technically sound?

Yes

Reviewer Expertise:

Entomolgy, genome, evolution

I confirm that I have read this submission and believe that I have an appropriate level of expertise to confirm that it is of an acceptable scientific standard.

# Reviewer report: Reviewer response for version 1

**DOI:** 10.21956/wellcomeopenres.24882.r94745

**Published:** 2024-08-26

**Authors:** Craig Wilding

**Affiliations:** 1 Liverpool John Moores University, Liverpool, UK

Obbard et al present an assembled genome sequence of *D. limbata*, one of approximately 30 *Drosophila* from the UK and Ireland (although it's distribution is wider). They provide a detailed (within the constraints of the DToL Genome Notes) introduction to this species and its ecology.

 The methods are thorough and provide links to all tools used.

 The final assembly is similar in size and completeness ((with a 97.4% BUSCO completeness) to other *Drosophila* sequenced by the DToL. Drosophilid species have four pairs of chromosomes including the sex chromosomes (males being XY).

 The 6 chromosomal psuedomolecules including the X chromosome that were scaffolded during this study do therefore not encapsulate the full typical karyotype of Drosophilids (although the authors report that the Y is likely in the unplaced contigs).

 My only comments are:

 1). The methods states that 'idDroLimb2 sample was weighed and dissected on dry ice'. What was the purpose of dissection here? It seems likely that the whole sample was homogenised and used for DNA extraction so why the dissection?

 2). I was not clear on Figure 3 what is being shown. How do I discern the data for this species? And when the legend says 'sequences are coloured by Phylum, how many phyla are included? what is 'total' and what is 'no hit'? This is undoubtedly my unfamiliarity with this kind of plot but the other legends are so clear and explanatory, that this one stands out as unclear (at least to me).

 3. Is the 'loss' of the Y chromosome to the unplaced contigs typical of other *Drosophila *assemblies or a peculiarity of this species? With the accumulation of *Drosophila* sequences (including in DToL) can anything be done with the tools used to improve the likelihood that the Y is successfully 'pulled out'?

Are sufficient details of methods and materials provided to allow replication by others?

Yes

Is the rationale for creating the dataset(s) clearly described?

Yes

Are the datasets clearly presented in a useable and accessible format?

Yes

Are the protocols appropriate and is the work technically sound?

Yes

Reviewer Expertise:

Genomics, population genetics, evolutionary biology

I confirm that I have read this submission and believe that I have an appropriate level of expertise to confirm that it is of an acceptable scientific standard.
